# Evaluations of the setup discrepancy between BrainLAB 6D ExacTrac and cone-beam computed tomography used with the imaging guidance system Novalis-Tx for intracranial stereotactic radiosurgery

**DOI:** 10.1371/journal.pone.0177798

**Published:** 2017-05-19

**Authors:** Se An Oh, Jae Won Park, Ji Woon Yea, Sung Kyu Kim

**Affiliations:** 1 Department of Radiation Oncology, Yeungnam University Medical Center, Daegu, Korea; 2 Department of Radiation Oncology, Yeungnam University College of Medicine, Daegu, Korea; North Shore Long Island Jewish Health System, UNITED STATES

## Abstract

The objective of this study was to evaluate the setup discrepancy between BrainLAB 6 degree-of-freedom (6D) ExacTrac and cone-beam computed tomography (CBCT) used with the imaging guidance system Novalis Tx for intracranial stereotactic radiosurgery. We included 107 consecutive patients for whom white stereotactic head frame masks (R408; Clarity Medical Products, Newark, OH) were used to fix the head during intracranial stereotactic radiosurgery, between August 2012 and July 2016. The patients were immobilized in the same state for both the verification image using 6D ExacTrac and online 3D CBCT. In addition, after radiation treatment, registration between the computed tomography simulation images and the CBCT images was performed with offline 6D fusion in an offline review. The root-mean-square of the difference in the translational dimensions between the ExacTrac system and CBCT was <1.01 mm for online matching and <1.10 mm for offline matching. Furthermore, the root-mean-square of the difference in the rotational dimensions between the ExacTrac system and the CBCT were <0.82° for online matching and <0.95° for offline matching. It was concluded that while the discrepancies in residual setup errors between the ExacTrac 6D X-ray and the CBCT were minor, they should not be ignored.

## Introduction

The clinical efficacy of intracranial stereotactic radiosurgery (SRS) on brain tumors has been previously established [1], [2], [3], [4], [5]. SRS has a relatively higher dose per fraction than conventional radiation therapy and should generally be administered in one fraction. Hypo-fractionated radiotherapy can be administered in 2–5 fractions if the size of the tumor is too large or the dose is above the limit radiation dose to surrounding normal organs [6], [7]. With sophisticated radiation treatment techniques such as SRS and hypo-fractionated radiotherapy, imaging guidance systems, such as ExacTrac (BrainLAB, Feldkirchen, Germany) and cone-beam computed tomography (CBCT; Varian Medical System, CA, USA; Elekta Oncology System, Crawley, UK) are indispensable, as they improve the accuracy of patient localization setup and tumor targeting in contouring [8], [9], [10], [11], [12], [13].

Since SRS is delivered with a large dose in a single fraction, it requires an extremely steep dose gradient so that minimal radiation is delivered to normal organs while maximal radiation is delivered to the tumor. In order to lower the radiation dose to normal organs, a minimal setup margin of the tumor must be established. On the other hand, a small setup margin established to reduce the dose of radiation reaching normal organs may result in poor clinical results due to under or over radiation dose delivered to the tumor itself, in addition to the increased uncertainties related to inter-fractional and intra-fractional setup errors.

For this reason, the role of imaging guidance systems such as ExacTrac and CBCT in intracranial SRS is hugely important. Due to a limited number of images, ExacTrac cannot provide as much visualization information as CBCT. However, ExacTrac can provide clinical benefits, including faster patient positioning, corrections using the 6D couch, motion tracking, and smaller radiation dose [10]. As of yet, it is unclear which of these two imaging guidance systems is more accurate, and the level of systemic discrepancy when using the ExacTrac and CBCT in the same patient has not been established.

To address these issues, a study by Ma et al. [10] evaluated 18 patients treated with fractionated stereotactic radiotherapy in the cranium; a CIRS (Computerized Imaging Reference System, Inc., Norfolk, VA) anthropomorphic head phantom compared the residual setup errors between 6 degree-of-freedom ExacTrac X-ray and CBCT. The residual setup error for the root-mean-square (RMS) value between the ExacTrac system and CBCT was <0.5 mm in the phantom and <1.5 mm in the patients for the translational direction, and <0.2° in the phantom and <1.0° in the patients for the rotational direction. They determined the difference in residual setup errors between the 6 DOF ExacTrac X-ray and CBCT were almost negligible. Ma et al. analyzed the difference in residual setup error between the two systems using only setup error data from 18 patients. However, Ma et al. mentioned about the limitation of the study for the small number of patients was enrolled and helpful for the additional study with more patients and more images. In the current study, we aimed to overcome this limitation.

Recently, several authors have reported the geometric accuracy of the 6D ExacTrac image guide system of the Novalis Tx in the frameless image-guide radiosurgery system for intracranial lesions. Lamba et al. [14] evaluated the hidden target test with the 0.7±0.5 mm for frame-based and the 0.6±0.2 mm for image-based intracranial radiosurgery. Also, the difference between frame-based and image-guided alignment for patients who underwent frame-based radiosurgery was 0.9±0.5 mm (anterior-posterior), -0.2±0.4 mm (superior-inferior), and 0.3±0.5 mm (left-right). Takakura et al. [15] assessed the overall geometric accuracy of ExacTrac system of the Novalis system in terms of the uncertainty using the head and neck phantom attached to the frameless radiosurgery system. The accuracy in positional correction with the robotic couch was 0.07±0.22 mm, and the overall geometric accuracy based on the concept of uncertainty in the intracranial SRS with the Novalis Tx was 0.31±0.77 mm. Ramakrishna et al. [16] demonstrated overall system accuracy using 57 hidden-target tests. Mean error magnitude indicates the 0.7 mm (SD = 0.3 mm) and mean deviation between frame-based and image-guided initial positioning was 1.0 mm (SD = 0.5 mm). The mean intra-fraction shift magnitude observed for the frame was 0.4 mm (SD = 0.3 mm) compared to 0.7 mm (SD = 0.5 mm) for the mask system.

Therefore, we decided that more data was needed to determine the residual setup error between the 6 DOF ExacTrac and CBCT imaging guidance systems. To this aim, we registered 107 patients treated with intracranial SRS in 1–4 fractions from August 2012 to July 2016 and for whom 6 DOF ExacTrac and CBCT were utilized for pretreatment verifications. The objective of this study was to evaluate the setup discrepancy between BrainLAB 6 DOF ExacTrac and CBCT with the imaging guidance system Novalis Tx in intracranial SRS, and to compare results with the residual setup errors reported by previous studies.

## Materials and methods

### Ethics statement

**Fig 1:**
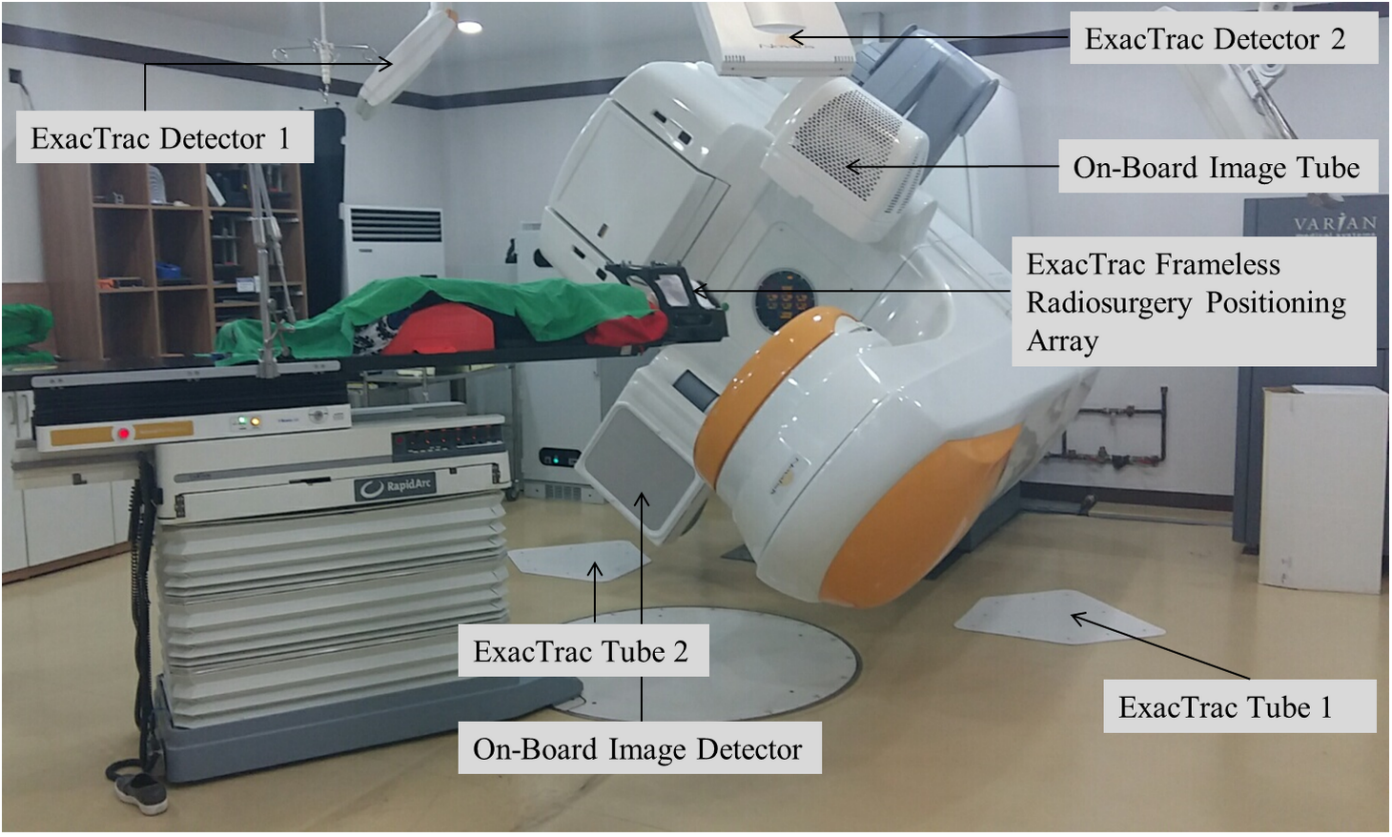
Image of the patient setup with ExacTrac frameless radiosurgery positioning array (BrainLAB AG, Feldkirchen, Germany).

This study was approved by the Institutional Review Board of Yeungnam University Medical Center (YUMC 2016-10-041), and patient consent was specially waived under the approval of the institutional review board because patient data were investigated anonymously. The individual pictured in Fig 1 of this manuscript provided written informed consent (as outlined in the PLOS consent form) to publish these case details.

A white stereotactic head frame mask (R408; Clarity Medical Products, Newark, OH) used to fix the head with the standard BrainLAB systems.

### Patient selection

A total of 107 consecutive patients for whom the white stereotactic head frame mask (R408; Clarity Medical Products, Newark, OH) was used to fix the head during intracranial SRS between August 2012 and July 2016 were included in this study. Table 1 outlines the patient characteristics and treatment details. The patients’ ages ranged from 16–81 years, with an average age of 58 years. The number of female patients was 50 (47%) and the number of male patients was 57 (53%). Pairs of 138 image verifications with 6 degrees-of-freedom ExacTrac X-ray and CBCT were performed for all 107 patients.

**Table 1:** Patient characteristics and treatments included.

Patient characteristics	
Number of patients	N = 107
Median age(Range)	58(16–81)
Gender(%)	
Female	50(47)
Male	57(53)
Treatment characteristics	
Number of treatment regions (%)	Number of Patients (%)/Number of Images (%)
1	83(77.6) / 83 (60.1)
2	19(17.8) / 38 (27.5)
3	3(2.8) / 9(6.5)
4	2(1.9) / 8 (5.8)
Sum	107 / 138
Techniques used for the treatment (%)	
DCAT	117(85)
IMRT	21(15)
Fraction schemes (dose/fraction) (%)	
21 Gy in 3 fractions (7 Gy)	1(1)
28 Gy in 4 fractions (7 Gy)	2(1)
27 Gy in 3 fractions (9 Gy)	1(1)
30 Gy in 3 fractions (10 Gy)	1(1)
12–23 Gy in a single fraction	133(96)

Abbreviations: IMRT = Intensity modulated radiotherapy; DCAT = Dynamic conformal arc therapy.

### Immobilization and CT simulation

A white stereotactic head frame mask (R408; Clarity Medical Products, Newark, OH) was used to fix the head with the standard BrainLAB systems. The stereotactic mask set was composed of 5 components including a top mask, middle mask, rear mask, package of loose pellets, and dental support stripe. Patients fixed by the stereotactic mask were scanned with 2-mm thickness using the BrainLAB^TM^ CT localizer frame with a Philips Brilliance Big Bore CT simulator (Philips Inc., Cleveland, OH). The total process time is usually around 1 hour in our institution, including mask molding and CT scanning.

### Treatment planning and delivery techniques

Magnetic resonance (MR) simulation images (2-mm thick) obtained for treatment planning were used in combination with planning CT. The MR simulation protocol of our institution utilizes 2-mm thick scans with T1-weighted axial MR images, T2-weighted axial MR images, and gadolinium-enhanced T1-weighted axial MR images. The BrainLAB iPlan RT Dose 4.5.1 (BrainLAB, Feldkirchen, Germany) treatment planning system was used for calculation with pencil beam algorithm for intracranial SRS. A total of 133 tumors were prescribed doses of 12–23 Gy delivered in a single fraction with various dose schedules (21 Gy in 3 fractions for 1 target, 28 Gy in 4 fractions for 2 targets, 27 Gy in 3 fractions for 1 target, and 30 Gy in 3 fractions for 1 target). Regarding techniques, 117 (85%) cases were treated with Dynamic Conformal Arc Therapy [17], and 21 (15%) cases were treated with intensity-modulated radiotherapy [8], [18]. Fig 1 depicts patient setup with ExacTrac Frameless Radiosurgery Positioning Array (BrainLAB AG, Feldkirchen, Germany). A white stereotactic head frame mask (R408; Clarity Medical Products, Newark, OH) was used to fix patients’ heads when using the standard BrainLAB system.

### Image registration and setup protocol

**Fig 2:**
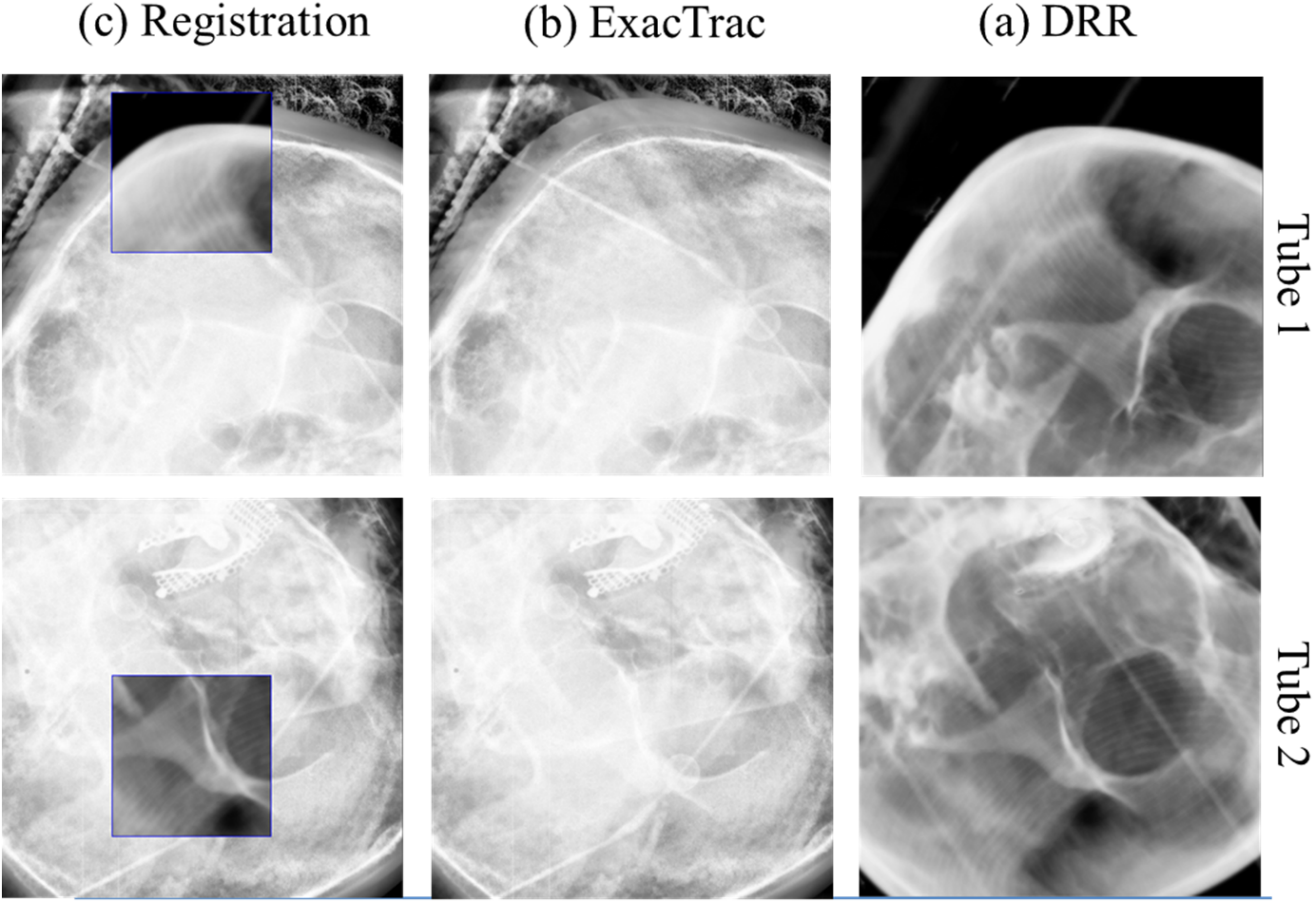
Image registration using kV tube 1 and tube 2 with the BrainLAB ExacTrac for patient #18. **(**a) DRR image; (b) ExacTrac image; and (c) image registration.

**Fig 3:**
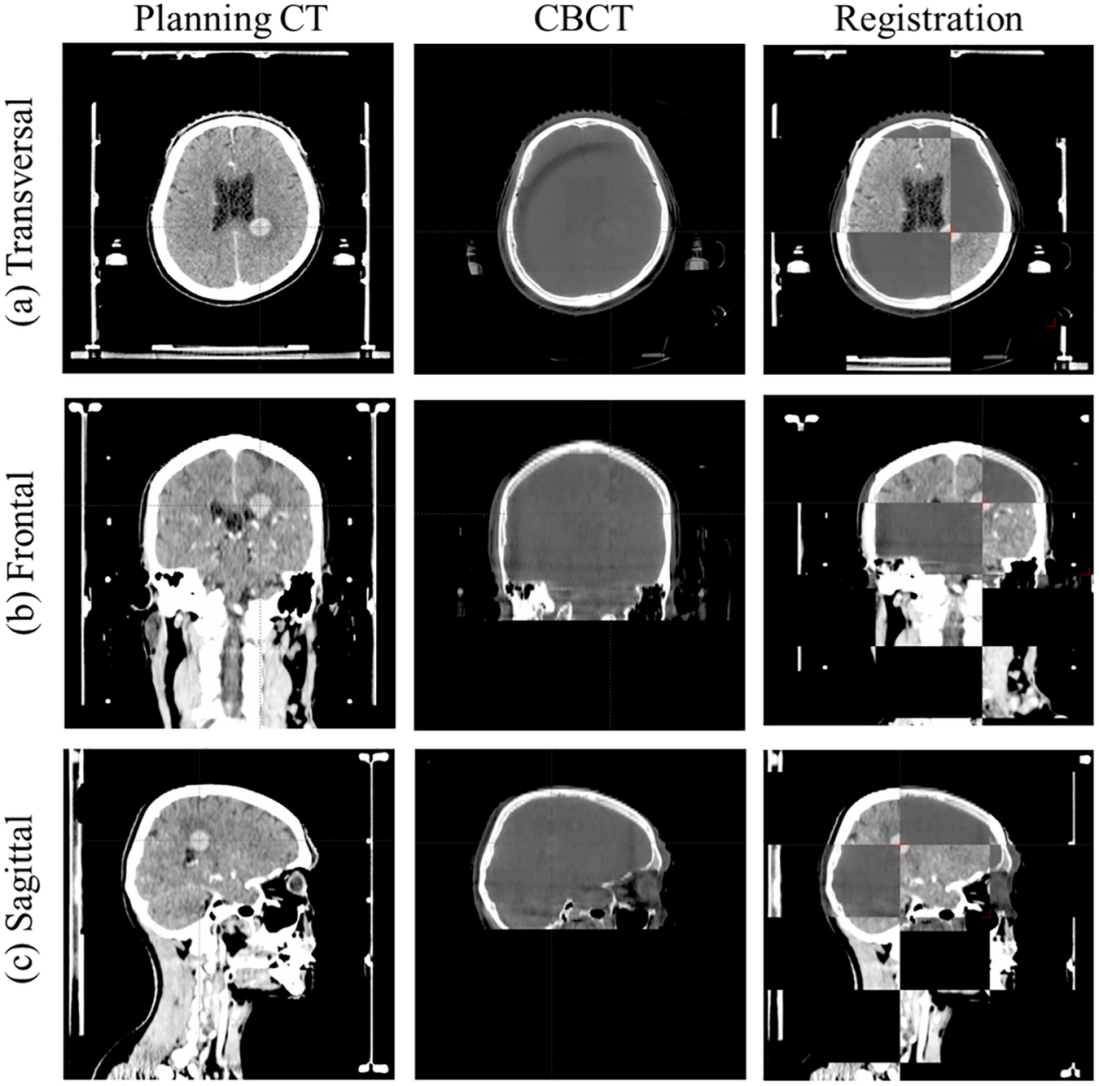
Image registration using planning computed tomography (CT) and cone-beam computed tomography (CBCT) for patient #18 with offline 6D fusion in offline review. (a) Axial planes; (b) Coronal planes; and (c) Sagittal planes.

The first setup images for all 107 patients were acquired with the 6D ExacTrac (BrainLAB AG, Feldkirchen, Germany) using the two floor-mounted kV X-ray tubes. Registration between the Digitally Reconstructed Radiograph (DRR) obtained from the simulation CT image and ExacTrac kV CT image was performed with bony anatomy matching based on the whole skull. Fig 2 shows the image registration using kV tube 1 and tube 2 with BrainLAB ExacTrac for patient #18 with the DRR image, ExacTrac image, and image at registration. Setup corrections with 6 degrees-of-freedom for the translational (lateral, longitudinal, and vertical) and rotational (pitch, roll, and yaw) dimensions were transferred to the BrainLAB robotic couch system. Subsequently, second setup images were acquired for setup verifications after the first setup corrections using the 6 DOF ExacTrac system. In accordance with our institution’s protocol of the image guidance system Novalis-Tx for intracranial SRS, the tolerance limit was set to 0.5 mm for the translations and 0.5° for the rotations. If the correction value is within the tolerance limit value with 0.5 mm for the translations and 0.5° for the rotations, there is not corrected. While the patients were immobilized in the same state as when the image was taken with the verification image using 6D ExacTrac, online 3D CBCT images were acquired from the rotation of the kV on-board-imager for the translational (lateral, longitudinal, and vertical) and rotational (yaw) dimensions. In addition, after radiation treatment, registration between the CT simulation images and the CBCT images was performed with offline 6D fusion in Offline review (ARIA 8.6; Varian Medical System, Palo Alto, CA), with the same parameters for patient position for the translational (lateral, longitudinal, and vertical) and rotational (pitch, roll, and yaw) dimensions. Fig 3 indicates the image registration using the planning CT and CBCT for patient #18 in the axial, coronal, and sagittal planes.

### Analysis of the residual setup errors between the ExacTrac and CBCT

The image registration among the 6D ExacTrac, 3D CBCT, 6D CBCT, and planning CT were analyzed with regard to the RMS and standard deviation of the residual setup errors in the translational (lateral, longitudinal, vertical) and rotational (pitch, roll, yaw) dimensions. Comparisons were made using paired t-tests, and all analyses were conducted using IBM SPSS Statistics (version 22; SPSS, Chicago, IL, USA). A P value of less than 0.05 was considered statistically significant.

## Results and discussion

**Fig 4:**
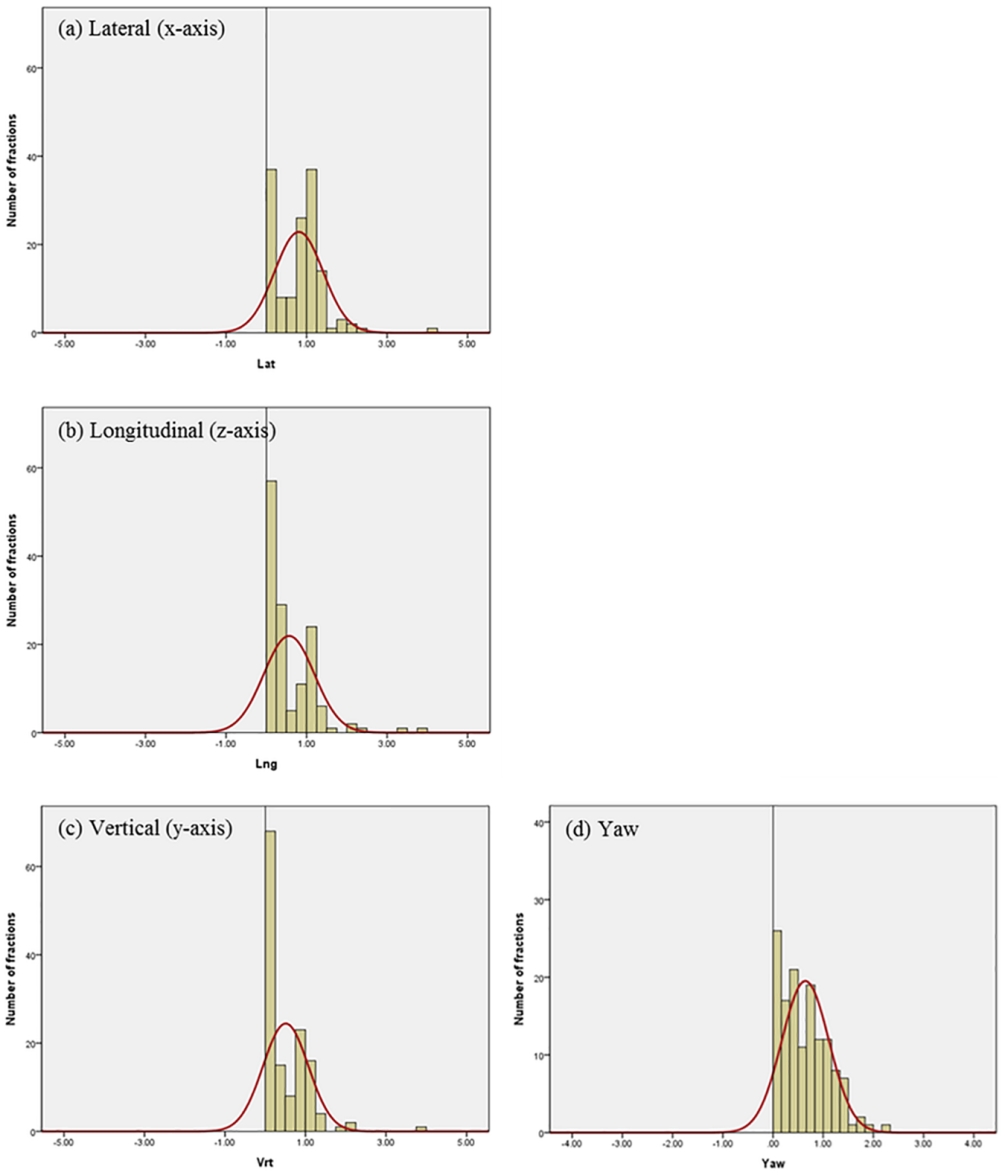
Histogram and normalized curves for the translational and rotational variations between the 6D ExacTrac and the 3D cone-beam computed tomography (CBCT). (a) Lateral (x-axis); (b) longitudinal (z-axis); (c) vertical (y-axis); and (d) yaw dimensions.

**Fig 5:**
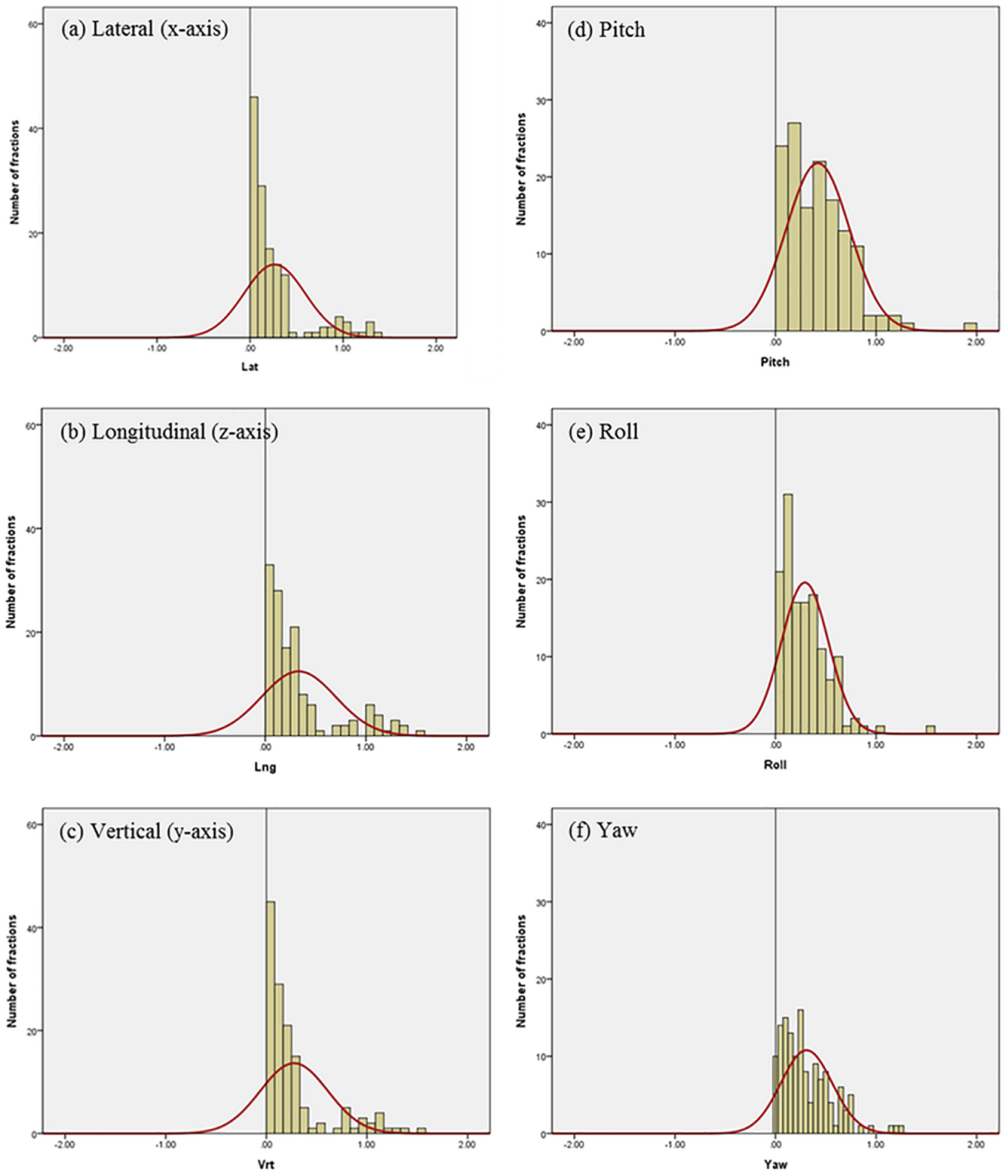
Histogram and normalized curves for the translational and rotational variations between the 6D ExacTrac and the 6D cone-beam computed tomography (CBCT). (a) Lateral (x-axis); (b) longitudinal (z-axis); (c) vertical (y-axis); (d) pitch; (e) roll; and (f) yaw dimensions.

**Fig 6:**
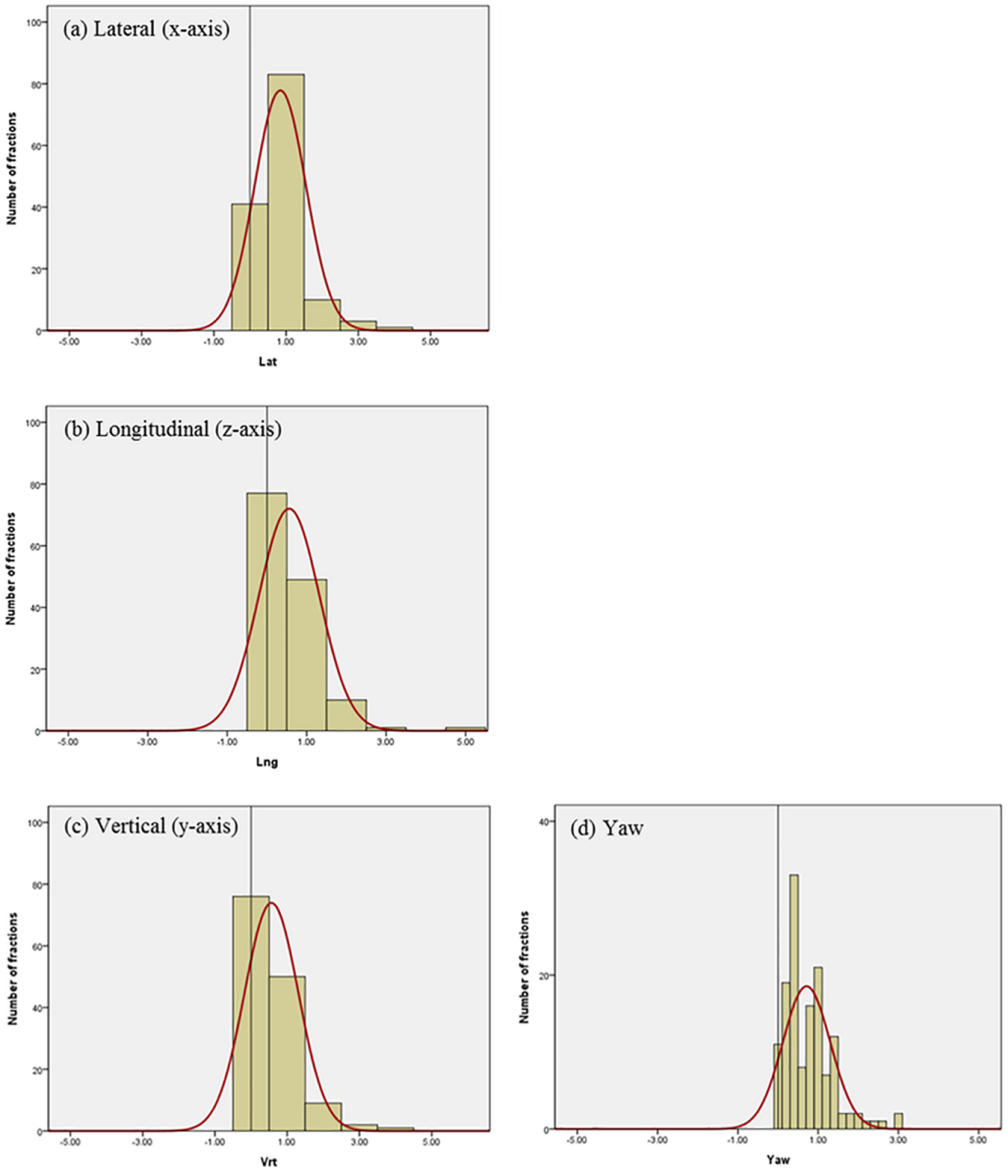
Histogram and normalized curves for the translational and rotational variations between the 3D CBCT and the 6D CBCT. (a) Lateral (x-axis); (b) Longitudinal (z-axis); (c) Vertical (y-axis), and (d) Yaw dimensions.

Differences in residual setup errors between the ExacTrac and CBCT were analyzed with 107 patients treated with intracranial SRS using a total of 138 pairs of scans in our image registrations and setup protocol. Fig 4 shows the histogram and normalized curves for the translational and rotational variations between the 6D ExacTrac and the 3D CBCT for the lateral (x-axis), longitudinal (z-axis), vertical (y-axis), and yaw dimensions. Fig 5 shows the histogram and normalized curves for the translational and rotational variations between the 6D ExacTrac and the 6D CBCT for the lateral (x-axis), longitudinal (z-axis), vertical (y-axis), pitch, roll, and yaw dimensions. Fig 6 shows the histogram and normalized curves for the translational and rotational variations between the 3D CBCT and the 6D CBCT for the lateral (x-axis), longitudinal (z-axis), vertical (y-axis), and yaw dimensions.

Table 2 lists the residual setup errors and differences among the 6D ExacTrac, 3D CBCT, and 6D CBCT. Residual setup errors reported by Ma et al. [10] and Chang et al. [12] were comparable to those found in our study. The factors in the translational dimension that were statistically significant were the lateral (x-axis) and vertical (y-axis) dimensions between the 6D ExacTrac and the 3D CBCT, the longitudinal dimension between the 6D ExacTrac and the 6D CBCT, and the lateral (x-axis) dimension between the 3D CBCT and the 6D CBCT. The factors in the rotational dimension that were statistically significant were pitch (x-axis) and roll (z-axis) dimensions between the 6D ExacTrac and the 6D CBCT. The RMS of the difference in the translational dimensions between the ExacTrac system and the CBCT were <1.01 mm for online matching and <1.10 mm for offline matching. In addition, the RMS of the difference in the rotational dimensions between the ExacTrac system and the CBCT were <0.82° for online matching and <0.95° for offline matching.

**Table 2:** Residual setup errors and differences among the 6D ExacTrac, 3D cone-beam computed tomography (CBCT), and 6D CBCT when used for intracranial stereotactic radiosurgery (N = 107, n = 138). Residual setup errors reported by Ma et al. [10] and Chang et al. [12] are also shown.

Series	Region	Number of patient	Directions	Setup error for 6D ExacTrac		Setup error for 3D CBCT		Setup error for 6D CBCT		6D ExacTrac vs 3D CBCT			6D ExacTrac vs 6D CBCT			3D CBCT vs 6D CBCT		
										Difference		p-Value of paired t-test	Difference		p-Value of paired t-test	Difference		p-Value of paired t-test
				RMS	SD	RMS	SD	RMS	SD	RMS	SD		RMS	SD		RMS	SD	
Ma et al. [10]	Brain	N = 18, n = 50	Translational															
			Lateral(x-axis)(mm)	0.47	0.47	0.76	0.74	0.83	0.81	0.65	0.63	0.646	0.71	0.70	0.219			
			Longitudinal(z-axis)(mm)	0.84	0.79	1.22	1.08	0.99	0.93	0.88	0.82	0.012^a^	0.76	0.71	0.028^a^			
			Vertical-(y-axis)(mm)	0.53	0.48	1.28	1.16	1.25	1.20	1.23	1.14	0.007^a^	1.30	1.22	0.002^a^			
			Rotational															
			Pitch(x-axis)(°)	0.36	0.35			0.39	0.38				0.27	0.26	0.275			
			Roll(z-axis)(°)	0.37	0.37			0.33	0.31				0.40	0.38	0.412			
			Yaw(y-axis)(°)	0.45	0.43	0.58	0.56	0.38	0.36	0.54	0.51	0.832	0.41	0.40	0.643			
Chang et al. [12]	Spine	N = 11, n = 16	Translational															
			Lateral(x-axis)(mm)							0.47	0.31	0.042^a^	0.59	0.60	0.042^a^	0.45	0.45	0.317
			Longitudinal(z-axis)(mm)							0.77	0.86	0.893	0.90	0.98	0.892	1.10	1.22	1.000
			Vertical-(y-axis)(mm)							1.23	0.72	0.043^a^	0.50	0.36	0.043^a^	0.63	0.55	0.157
			Rotational															
			Pitch(x-axis)(°)							0.91	0.87	0.686	0.53	0.41	0.080	0.92	0.41	0.892
			Roll(z-axis)(°)							1.27	1.27	0.345	0.84	0.43	0.043^a^	0.84	0.43	0.686
			Yaw(y-axis)(°)							0.73	0.17	0.043^a^	0.54	0.49	0.042^a^	0.54	0.49	0.257
Our Study	Brain	N = 107, n = 138	Translational															
			Lateral(x-axis)(mm)	0.20	0.20	0.97	0.65	0.36	0.36	1.01	0.60	<0.001^a^	0.42	0.33	0.803	1.10	0.71	<0.001^a^
			Longitudinal(z-axis)(mm)	0.24	0.24	0.77	0.77	0.41	0.39	0.84	0.63	0.425	0.49	0.37	0.001^a^	0.94	0.76	0.282
			Vertical-(y-axis)(mm)	0.20	0.20	0.76	0.75	0.37	0.37	0.76	0.56	0.028^a^	0.43	0.34	0.970	0.93	0.74	0.043^a^
			Rotational															
			Pitch(x-axis)(°)	0.18	0.18			0.51	0.40				0.53	0.32	<0.001^a^			
			Roll(z-axis)(°)	0.17	0.17			0.37	0.33				0.37	0.23	<0.001^a^			
			Yaw(y-axis)(°)	0.22	0.22	0.80	0.80	0.31	0.31	0.82	0.49	0.226	0.40	0.26	0.181	0.95	0.62	0.566

Abbreviations: N = number of patients; n = number of scans.

^a^ p < 0.05.

**Fig 7:**
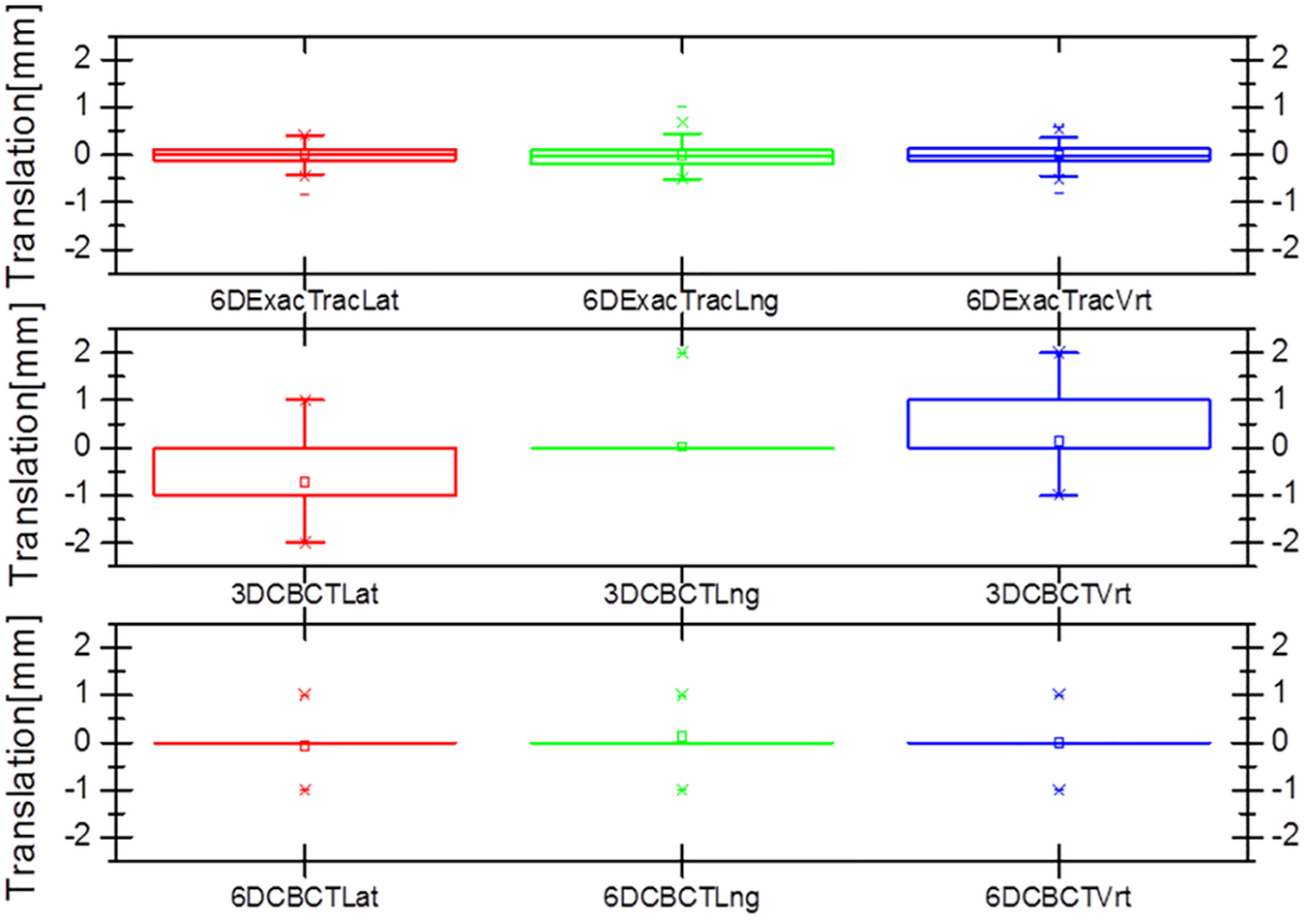
Box and whisker plots of the translational variations in the lateral, longitudinal, and vertical directions with 6D ExacTrac, 3D cone-beam computed tomography (CBCT), and 6D CBCT.

**Fig 8:**
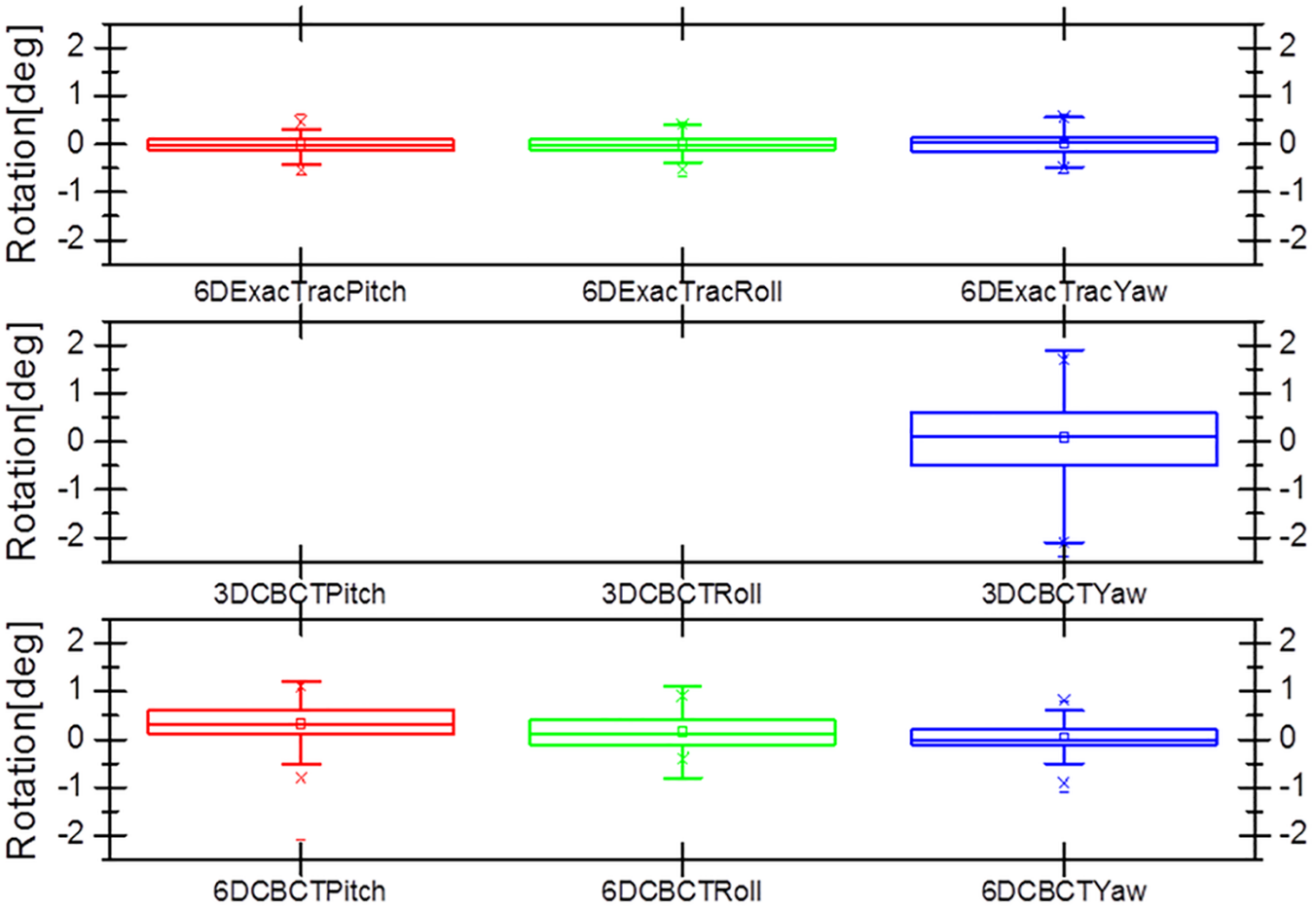
Box and whisker plots of the rotational variations in the pitch, roll, and yaw directions with 6D ExacTrac, 3D cone-beam computed tomography (CBCT), and 6D CBCT.

**Fig 9:**
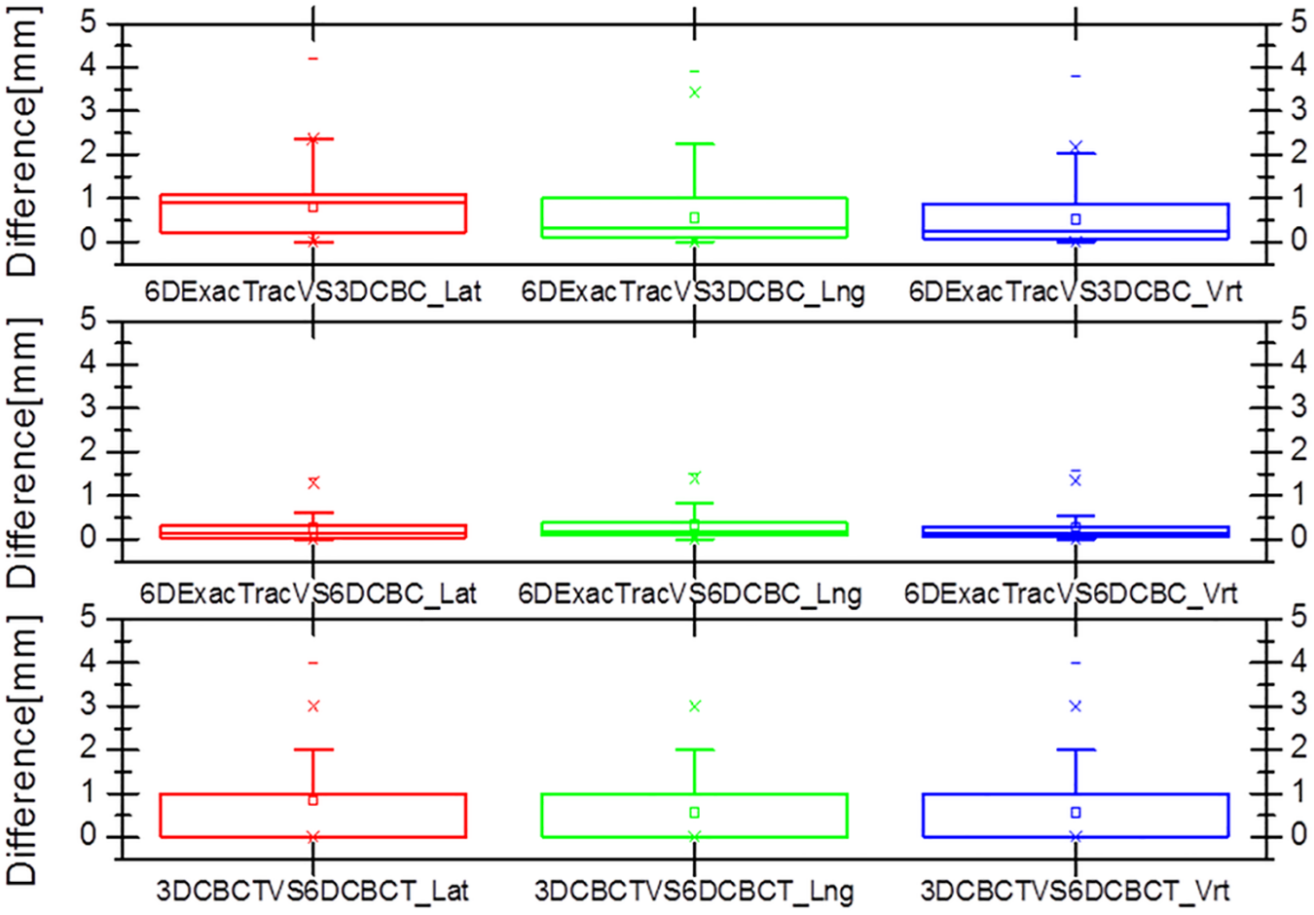
Box and whisker plots of the translational difference variations in the lateral, longitudinal, and vertical directions for the 6D ExacTrac vs. 3D cone-beam computed tomography (CBCT), 6D ExacTrac vs. 6D CBCT, and 3D CBCT vs. 6D CBCT.

**Fig 10:**
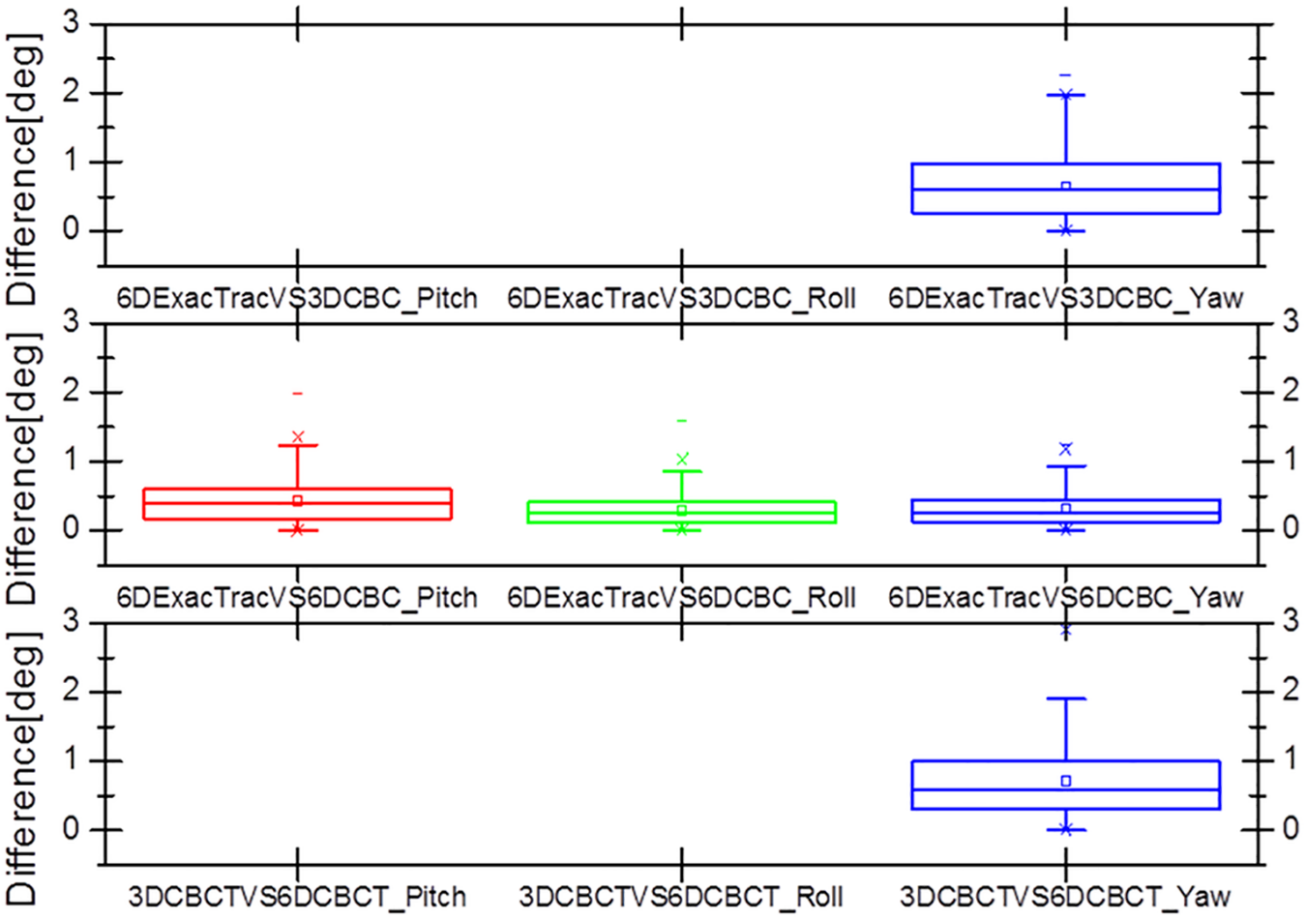
Box and whisker plots of the rotational difference variations in the pitch, roll, and yaw directions for the 6D ExacTrac vs. 3D cone-beam computed tomography (CBCT), 6D ExacTrac vs. 6D CBCT, and 3D CBCT vs. 6D CBCT.

Figs 7 and 8 show box and whisker plots of the translational variations in the lateral, longitudinal, and vertical directions, and of the rotational variations in the pitch, roll, and yaw dimensions with 6D ExacTrac, 3D CBCT, and 6D CBCT. Fig 9 shows the box and whisker plots of the translational difference variations in the lateral, longitudinal, and vertical directions for the 6D ExacTrac and 3D CBCT, 6D ExacTrac and 6D CBCT, and 3D CBCT and 6D CBCT. Fig 10 shows the box and whisker plots of the rotational difference variations in the pitch, roll, and yaw dimensions for the 6D ExacTrac and 3D CBCT, 6D ExacTrac and 6D CBCT, and 3D CBCT and 6D CBCT.

In a similar study, Ma et al. [10] compared the residual setup errors between the ExacTrac X-ray 6 degree-of-freedom and CBCT for a head phantom and 18 patients receiving intracranial non-invasive fractionated SRS. The RMS of the differences for translations was typically <0.5 mm for the phantom and <1.5 mm for the patients. The RMS of the differences for rotation was <0.2° for the phantom and <1.0° for the patients. The RMS of the difference of the translational and the rotational variation between the ExacTrac and the CBCT thus coincided well with our results. One of the potential sources for the discrepancies observed between BrainLAB 6D ExacTrac and CBCT might be the inter-scan between the BrainLAB 6D ExacTrac and CBCT for the patient motion. Ma et al. used the U-frame mask (BrainLAB, Heimstetten, Germany) to immobilize and can occur the intra-fraction motion to <0.5 mm in cranial cases. Our study was able to reduce the intra-fraction variation by using the white stereotactic head frame masks (R408; Clarity Medical Products, Newark, OH), thus minimizing this potential source of discrepancies found between BrainLAB 6D ExacTrac and CBCT.

Another potential source of discrepancies between the two systems is intra-fractional variation, which describes the movements of the patient with the mask during radiation treatment. Spadea et al. [19] investigated the intra-fraction setup variability using the IR optical localization and stereoscopic kV X-ray imaging in ExacTrac X-ray 6D system. The size of intra-fraction motion was (median ± quartile) 0.3 ± 0.3 mm for optical localization, and 0.9 ± 0.8 mm for X-ray images. To illustrate the discrepancies between BrainLAB 6 DOF ExacTrac and CBCT, we need to consider the intrinsic accuracy of the two systems.

Chang et al. [12] evaluated the residual setup errors between the ExacTrac X-ray 6D and CBCT for an anthropomorphic phantom and 16 patients treated with spinal non-invasive stereotactic body radiation therapy. Phantom experiments indicated that translational and rotational discrepancies in the RMS were <1.0 mm and <1°, respectively. The patients’ results showed translational and rotational discrepancies in the RMS were <2.0 mm and <1.5°, respectively. Compared to intracranial SRS, the results of the RMS for the differences for translational and rotational discrepancies in spinal stereotactic body radiation therapy were slightly higher.

One limitation of the present study is that we only evaluated the difference between the ExacTrac 6D X-ray and CBCT imaging in intracranial SRS. If we had included treatment of extra-cranial regions, discrepancies in the residual setup errors between ExacTrac X-ray 6D and CBCT may also have been introduced. Thus, in future work, we plan to evaluate the residual setup errors between the ExacTrac 6D X-ray and the CBCT when used in treating extra-cranial regions.

## Conclusions

This study evaluated the residual setup errors between ExacTrac 6D X-ray and CBCT when used to treat 107 patients with intracranial SRS. While the discrepancies of the residual setup errors between the ExacTrac 6D X-ray and the CBCT were minor, they should not be ignored.

## References

[1] Andrews DW, Scott CB, Sperduto PW, Flanders AE, Gaspar LE, et al (2004) Whole brain radiation therapy with or without stereotactic radiosurgery boost for patients with one to three brain metastases: phase III results of the RTOG 9508 randomised trial. The Lancet 363: 1665–1672. 10.1016/S0140-6736(04)16250-8 15158627

[2] Aoyama H, Shirato H, Tago M, Nakagawa K, Toyoda T, et al (2006) Stereotactic radiosurgery plus whole-brain radiation therapy vs stereotactic radiosurgery alone for treatment of brain metastases: a randomized controlled trial. Jama 295: 2483–2491. doi: 10.1001/jama.295.21.2483 16757720 10.1001/jama.295.21.2483

[3] Kim J, Wen N, Jin J-Y, Walls N, Kim S, et al (2012) Clinical commissioning and use of the Novalis Tx linear accelerator for SRS and SBRT. Journal of Applied Clinical Medical Physics 13. 10.1120/jacmp.v13i3.3729 PMC5716565 22584170

[4] Yu J, Choi JH, Ma SY, Jeung T (2015) Outcomes after Reirradiation for Brain Metastases. Progress in Medical Physics 26: 137–142.

[5] Won YK, Lee JY, Kang YN, Jang JS, Kang J-H, et al (2015) Stereotactic radiosurgery for brain metastasis in non-small cell lung cancer. Radiation oncology journal 33: 207–216. doi: 10.3857/roj.2015.33.3.207 26484304 10.3857/roj.2015.33.3.207 PMC4607574

[6] Benedict SH, Yenice KM, Followill D, Galvin JM, Hinson W, et al (2010) Stereotactic body radiation therapy: the report of AAPM Task Group 101. Medical physics 37: 4078–4101. doi: 10.1118/1.3438081 20879569 10.1118/1.3438081

[7] Eaton BR, La Riviere MJ, Kim S, Prabhu RS, Patel K, et al (2015) Hypofractionated radiosurgery has a better safety profile than single fraction radiosurgery for large resected brain metastases. Journal of neuro-oncology 123: 103–111. doi: 10.1007/s11060-015-1767-4 25862006 10.1007/s11060-015-1767-4

[8] Oh SA, Kang MK, Kim SK, Yea JW (2013) Comparison of IMRT and VMAT techniques in spine stereotactic radiosurgery with international spine radiosurgery consortium consensus guidelines. Progress in Medical Physics 24: 145–153.

[9] Oh SA, Yea JW, Kang MK, Park JW, Kim SK (2016) Analysis of the setup uncertainty and margin of the daily exactrac 6D image guide system for patients with brain tumors. PloS one 11: e0151709 doi: 10.1371/journal.pone.0151709 27019082 10.1371/journal.pone.0151709 PMC4809593

[10] Ma J, Chang Z, Wang Z, Wu QJ, Kirkpatrick JP, et al (2009) ExacTrac X-ray 6 degree-of-freedom image-guidance for intracranial non-invasive stereotactic radiotherapy: comparison with kilo-voltage cone-beam CT. Radiotherapy and Oncology 93: 602–608. doi: 10.1016/j.radonc.2009.09.009 19846229 10.1016/j.radonc.2009.09.009

[11] Oh Y-K, Baek J, Kim O-B, Kim J-H (2014) Assessment of setup uncertainties for various tumor sites when using daily CBCT for more than 2200 VMAT treatments. Journal of Applied Clinical Medical Physics 15. 10.1120/jacmp.v15i2.4418 PMC5875470 24710431

[12] Chang Z, Wang Z, Ma J, O’Daniel JC, Kirkpatrick J, et al (2010) 6D image guidance for spinal non-invasive stereotactic body radiation therapy: Comparison between ExacTrac X-ray 6D with kilo-voltage cone-beam CT. Radiotherapy and Oncology 95: 116–121. doi: 10.1016/j.radonc.2009.12.036 20122747 10.1016/j.radonc.2009.12.036

[13] Infusino E, Trodella L, Ramella S, D’Angelillo RM, Greco C, et al (2015) Estimation of patient setup uncertainty using BrainLAB Exatrac X-Ray 6D system in image-guided radiotherapy. Journal of Applied Clinical Medical Physics 16. 10.1120/jacmp.v16i2.5102 PMC5690103 26103179

[14] Lamba M, Breneman JC, Warnick RE (2009) Evaluation of image-guided positioning for frameless intracranial radiosurgery. International Journal of Radiation Oncology* Biology* Physics 74: 913–919. 10.1016/j.ijrobp.2009.01.008 19327898

[15] Takakura T, Mizowaki T, Nakata M, Yano S, Fujimoto T, et al (2009) The geometric accuracy of frameless stereotactic radiosurgery using a 6D robotic couch system. Physics in medicine and biology 55: 1. 10.1088/0031-9155/55/1/001 19949261

[16] Ramakrishna N, Rosca F, Friesen S, Tezcanli E, Zygmanszki P, et al (2010) A clinical comparison of patient setup and intra-fraction motion using frame-based radiosurgery versus a frameless image-guided radiosurgery system for intracranial lesions. Radiotherapy and Oncology 95: 109–115. doi: 10.1016/j.radonc.2009.12.030 20116123 10.1016/j.radonc.2009.12.030

[17] Molinier J, Kerr C, Simeon S, Ailleres N, Charissoux M, et al (2016) Comparison of volumetric-modulated arc therapy and dynamic conformal arc treatment planning for cranial stereotactic radiosurgery. Journal of Applied Clinical Medical Physics 17. 10.1120/jacmp.v17i1.5677 PMC5690199 26894335

[18] Park JM, Park S-Y, Wu H-G, Kim J-i (2016) Treatment Plan Delivery Accuracy of the ViewRay System in Two-Headed Mode. Progress in Medical Physics 27: 169–174.

[19] Spadea MF, Tagaste B, Riboldi M, Preve E, Alterio D, et al (2011) Intra-fraction setup variability: IR optical localization vs. X-ray imaging in a hypofractionated patient population. Radiation oncology 6: 38 doi: 10.1186/1748-717X-6-38 21496255 10.1186/1748-717X-6-38 PMC3096920

